# Aging brains and waning clocks on the process of habituation

**DOI:** 10.18632/aging.100154

**Published:** 2010-06-03

**Authors:** Kristin Eckel-Mahan, Paolo Sassone-Corsi

**Affiliations:** University of California at Irvine, Irvine, CA 92697, USA

**Keywords:** 05/30/10; accepted: 06/02/10; published on line: 06/03/10

Cyclical patterns of activity that occur every twenty-four hours are referred to as circadian oscillations. Most organisms experience circadian oscillations of some kind and human biology sustains numerous circadian oscillations that support complex physiological functions. As we age, the circadian clock begins to break down. For example, the molecular rhythmicity in expression or activity of some proteins dampens and our sleep/wake cycles become disrupted. This waning clock may have numerous consequences on our cognitive processes including learning and memory. That the circadian clock influences learning and memory has been demonstrated repeatedly, however, how individual proteins that comprise the clock machinery contribute to learning and memory is not clear [1]. In the May issue of Aging, Kondratova et al. use mice genetically altered in the Clock, Bmal1, and the Cryptochrome (Cry1 and Cry2) genes to address what properties these circadian clock components impart to these cognitive processes [2].

While the brain assists in synchronization of peripheral clocks, to keep accurate time in the brain as well as the periphery, the circadian clock relies on a central transcriptional and translational feedback loop [3]. In brief, the transcription factors Clock and Bmal1 heterodimerize and activate target gene promoters via E boxes, short motifs generally found in the promoters of many oscillating genes. Two of these target genes include the Per and Cry genes, which dimerize and interact with Clock and Bmal1, to inhibit subsequent Clock:Bmal1-mediated gene transcription. While these proteins are expressed in most tissues of the body, including the hippocampus, how they participate in normal hippocampal function or neuronal plasticity is unclear. Using circadian mutant mice, the study of Kondratova et al. reveals a novel role for circadian clock genes, as modulators of adaptation and habituation [2].

Habituation, a form of non-associative learning, occurs when an organism reduces or ceases its response to a specific stimulus after prolonged or repeated exposure to that stimulus. Humans habituate to their surroundings constantly. Even early in human development, visual habituation can be easily observed. Repeatedly expose an infant to the same object and he loses interest, but when confronted with a novel object he will increase his visual attention to the new object- at least for a time. Kondratova et al. reveal that Bmal1-/- mice not only display hyperactivity in a novel environment but that they fail to habituate normally to that novel environment both within a session and between separate session. (While anxiety levels could certainly contribute to this phenotype, the study excludes this possibility with additional behavioral tests.) These results indicate that both short term and long term memory may be impaired in these animals. Perhaps contributing to this cognitive phenotype, Bmal1-/- mice age rapidly and show a corresponding increase in ROS in several parts of the body including the brain [2]. Interestingly, while clock mutant (clockΔ19) mice show a similar impairment in intersession habitutation (but not intrasession habituation), the arrhythmic Cry1,2-/- mice actually show a facilitation in habituation.

The use of global knockouts leaves the direct link between function of specific circadian clock proteins and habituation somewhat abstruse in the sense that the changes observed in habituation between the Bmal1-/- and Cry1,2-/- mice arise from large networks of neurons lacking these proteins. As habituation to novel environments involves participation of the prefrontal cortex and the hippocampus, the effects of these proteins on short and long term memory in localized brain regions remain beyond interpretation. SCN lesions have also been reported to impair rodent habituation [4], however, what this study convincingly reveals is that the contribution of specific circadian clock proteins to the process of habituation must be specific. While the Bmal1-/-, Cry1,2-/-, and ClockΔ19 mice used in the study are all arrhythmic, the absence of individual circadian proteins produces different effects on habituation, underscoring the idea that the roles of these proteins in habituation are not merely restricted to circadian timekeeping but rather, they govern synaptic plasticity in unique ways. For example, the neurotransmitter, acetylcholine oscillates in a circadian fashion in the brain and its apparent decline in the hippocampus during the process of habitation[5], may mean that the time of day gates an organism's response to novel and familiar inputs. The disparate behavior in the circadian mutant animals, however, implies that the contribution of distinct clock proteins to this cognitive process extends beyond that of local timekeeping in the brain. It is tempting to speculate that the aging Bmal1-/- brain contributes to the impairments in habituation. When more spatially and temporally restricted CNS expression is attained, however, it will be clearer exactly how and where Bmal1 encroaches on this process.
